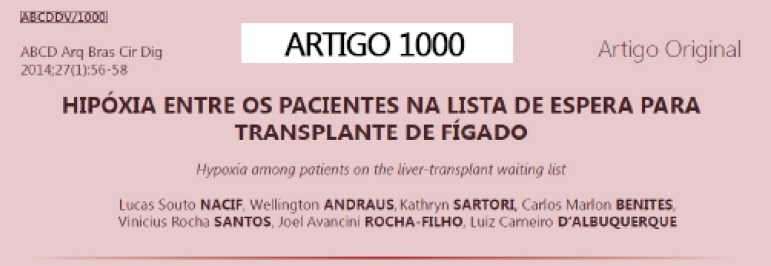# ABCD - BRAZILIAN ARCHIVES OF DIGESTIVE SURGERY: 1000 ARTICLES AND A HISTORY OF VICTORIES!

**DOI:** 10.1590/S0102-67202014000200001

**Published:** 2014

**Authors:** Osvaldo MALAFAIA, Nelson Adami ANDREOLLO, Bruno ZILBERSTEIN, Ivan CECCONELLO

What is the feeling of the editors of a medical journal when it reaches the significant mark of 1,000 published new scientific articles?

On this occasion, the editors and reviewers looking back about what meant these 1,000 papers published in the period of 27 years, from 1986 to 2013, confirms that they worked very hard to reach this target. Let suppose, if each article has at least 1,000 words, 1,000 articles addressing all possible issues related to digestive surgery will reach a million words, and this amount certainly corresponds to an encyclopedia!

The first article published in 1986 was written by the creator and founder of the Brazilian Archives of Digestive Surgery (ABCD), Prof. Dr. Henrique Walter Pinotti, about surgeonpatient relationship^1^. And in the presentation of a new journal he emphasized that: "This surgical breakthrough resulted in the publication of many scientific articles and editing books and journals. But even so, due to the majority of surgical patients are in Gastrointestinal Surgery area, there are few specialized sources to disseminate up the new information. For some time we have been considering publishing a journal dedicated to this specialty, with articles by Brazilian and foreign authors to be distributed abroad, as well as in Brazil. Since English is commonly used in medical and scientific specialties, would be published in both languages".

Professor Pinotti, in his speech at the beginning of the activities of the Brazilian College of Digestive Surgery (CBCD ) in 1988 emphasized that : "With education we not only inform, but we forge high level professionals, which can develop their knowledge, applying correctly their resources and thus able to benefit the patient. Knowing the limits of their competence, he will be able also to serve his patient avoiding the worst result of surgery, which is the iatrogenesis. Our College, in education, should develop the spirit that every person who wants to teach must have, and also finding the ones that want to learn. And the success achieved in the safe training can constitute stimuli for new frontiers of knowledge".

During all these years, since the publication of the first issue of the ABCD, many changes and advances occurred in medicine in general, in the digestive surgery and general surgery; new methods have emerged, in the preoperative and the postoperative care and also in the relationship between surgeon-patient^2^. And, undoubtedly, both ABCD and CBCD opened new doors to knowledge and have contributed to medical education in the country.

In 2009 the journal was accepted and included in the list of national journals indexed in SciELO (Scientific Electronic Library Online), came to be published in Portuguese and English, getting so more visibility, more value and, as already is accessed online, is visible for consultation worldwide through internet, thus raising its impact. In addition, in 2010 also was made an agreement with other surgical associations, and ABCD also became the official organ for scientific publication of the Brazilian Gastric Cancer Association, the Brazilian Chapter of the International Hepato- Biliary-Pancreato Association, Study Group of Pancreatic Diseases and the Brazilian Society of Metabolic and Bariatric Surgery^3^. Later, in 2013, the Sobracil (Brazilian Society of Videosurgery), was another association that came to incorporate the ABCD as its official journal for scientific papers.

From 2012, it was with great pride that the publishers of the ABCD communicated to all members and researchers of the related areas (Digestive Surgery, Gastroenterology, Endoscopy, Digestive Motility, Hepatology, Bariatric Surgery, Videosurgery and others) its inclusion in the MEDLINE/ PUBMED. Therefore, the journal will be a Brazilian vehicle to compete with the best journal in the world. ABCD, with these indexations, can positively contribute with the international ranking of Brazilian publications, climbing one more step in the number of publications on world^4^.

The feeling is of great satisfaction and accomplishment towards Brazilians surgeons, to members of the Brazilian College of Digestive Surgery and to the society in general. A journal that published 1,000 papers, all submitted to review of their referees and editors in regard to their ethical and scientific content, bilingual writing - Portuguese and English -, with results and conclusions, with good recommendations and with orientative final messages, certainly, have reached its maturity. We understand that the journal got greater visibility and even greater national and international accreditation; got higher quality in intellectual production; the authors were more valued in their scientific field and, as a result, they submitted higher number of papers to ABCD, contributing to increase overall national publications.

In this opportunity we have to thank the authors and coauthors who believed and sent their articles to us, contributing to the dissemination of this fantastic and important amount of knowledge with quality and to the ones who believed in the progress of the journal. We have to thank the Postgraduate Programs (strict sense) recommended by CAPES, who submitted their selected studies for publication and to all the editors and researchers who, directly or indirectly, contributed to the journal be even better. We must also to thank the graphic publisher Comunicare that contributed with professionalism and expertise on printing and finishing most part of ABCD existence, as well as the graphic design made by Bruno Ariede cannot be forgotten in this celebration.

In 2014, we will start the electronic submission and internal flow online of the articles, and a new website specially created to the journal, giving more professionalism to the whole process and facilitating the authors.

Finally, we must dream to aspire growing, to achieve victories and success; to do so, all of us must have a lot of dedication, great effort and, importantly, with a strong work team!

## Figures and Tables

**Figure:**